# Comparison of the Physicochemical Properties of Carboxymethyl Agar Synthesized by Microwave-Assisted and Conventional Methods

**DOI:** 10.3390/gels8030162

**Published:** 2022-03-04

**Authors:** Bo Qi, Shaoling Yang, Yongqiang Zhao, Yueqi Wang, Xianqing Yang, Shengjun Chen, Yanyan Wu, Chuang Pan, Xiao Hu, Chunsheng Li, Lunan Wang

**Affiliations:** 1 Key Laboratory of Aquatic Product Processing, Ministry of Agriculture and Rural Affairs, National R&D Center for Aquatic Product Processing, South China Sea Fisheries Research Institute, Chinese Academy of Fishery Sciences, Guangzhou 510300, China; qibo780210@163.com (B.Q.); wangyueqi@scsfri.ac.cn (Y.W.); yxqgd@163.com (X.Y.); chenshengjun@scsfri.ac.cn (S.C.); wuyygd@163.com (Y.W.); silverpfoxc@hotmail.com (C.P.); hnhuxiao@163.com (X.H.); lichunsheng@scsfri.ac.cn (C.L.); wln18616829169@163.com (L.W.); 2 Collaborative Innovation Center of Provincial and Ministerial Co-Construction for Seafood Deep Processing, Dalian Polytechnic University, Dalian 116034, China; 3 Co-Innovation Center of Jiangsu Marine Bio-Industry Technology, Huaihai Institute of Technology, Lianyungang 222005, China; 4 Hangzhou PuYu Technology Development Co., Ltd., Hangzhou 311300, China

**Keywords:** carboxymethyl agar, physicochemical characterization, microwave-assisted synthesis

## Abstract

The microwave-assisted carboxymethylation of agar to improve its physicochemical properties was investigated. Microwave power, reaction time, and temperature, ethanol concentration, and amounts of chloroacetic acid and sodium hydroxide were assessed for their effects on synthetic yield and degree of substitution (DS). All factors were positively correlated with DS within a certain range. Using optimized conditions, samples with different DS were prepared, and the physicochemical properties of unmodified and carboxymethyl agars prepared by microwave and conventional methods were compared. Carboxymethylation significantly changed the physicochemical properties of the agar, improving gel transparency and reducing dissolution temperature, gel strength, gel hardness, molecular weight, and molecular size; DS was the key factor. Specifically, higher DS values resulted in greater changes. The microwave-assisted method significantly shortened the reaction time and preserved molecular weight, gel strength, and texture hardness of the agar. Therefore, as an environmentally friendly method, microwave-assisted synthesis shows great promise for producing carboxymethyl agar.

## 1. Introduction

Agar is one of the oldest polysaccharide mixtures and is extracted from marine red algae of the orders Gelidiales and Gracilariales. It is widely used in foods, drinks, and medicine, especially in microbiological media and in tissue engineering, because of its unique gelling power, gel reversibility, and high hysteresis [1,2,3]. However, with the increasing diversification and specialization of agar applications, natural agar may not be ideal because of its large molecular weight, poor solubility in cold water, long dissolution time, and insufficient transparency in solutions and gels. Therefore, in recent years, there has been an increase in the demand for agar matrices with improved performance. For example, low-melting-point instant agar is required for cold beverages and food [4], highly transparent and clear agar is required for microbiological testing [5], and agar with improved shear stability is needed in multi-material mixture processing [6]. Therefore, modification methods are required to improve the physical and chemical properties of agar and broaden its applications.

Several studies [7,8] have shown that the physicochemical and functional properties, as well as the stability of agar, can be improved by replacing the hydroxyl groups with other functional groups, thus decreasing the dissolution temperature, improving the gel strength and transparency, and yielding gels tailored for particular industries. The most investigated chemical modifications of agar involve acetylation, hydroxypropylation, etherification, and oxidation [9]. Xiao et al. (2020) [10] modified agar through desulfation with hydrogen peroxide to significantly increase gel strength, whiteness, and transparency. Zhang et al. (2018) [5] modified agarose through oxyalkylation to significantly decreased gelling temperature and gel strength. Xiao et al. (2019) [11] successfully modified agar using octenyl succinic anhydride to obtain a lower gelling and melting temperature and a higher transparency. Cao et al. (2014) [12] modified agar through carboxymethylation that caused a significant decrease in the dissolving temperature, gelling temperature, gel strength, etc. Our research team primarily studied three modification methods, i.e., carboxymethylation, hydrogen peroxide oxidation, and hydroxypropylation, and compared their effects on agar properties [13]. We found that all three modified methods significantly increased the transparency of agar, whereas only carboxymethylation decreased its whiteness and thermal stability, in contrast to oxidation and hydroxypropylation.

In the numerous literature reports on various modification techniques, carboxymethylation modification is seldom used. However, carboxymethylation modification significantly improves the solubility, transparency, and colloidal system stability of polysaccharides, particularly their solubility [14]. Microwave-assisted carboxymethylation is an efficient, novel, and environmentally friendly polymer synthesis technology that has drawn significant attention in recent years [15]. Notably, the microwave-assisted method rapidly applies a uniform heat energy to a reaction, effectively improving the rate of synthesis [16]. Compared to traditional methods, the microwave-assisted method has the advantages of short reaction time, high efficiency, and good product homogeneity [17]. It has been applied to modify starch [18]. Some unexpected and interesting results have been obtained during its applications. Nevertheless, there limited research has been conducted on its use for agar modification. Like starch, agar is also widely used in many fields of production and life, and there is a need to constantly improve its physicochemical properties to meet new requirements in different fields. Based on our previous research, in order to further refine the effects of carboxymethylation on agar and improve its performance in new applications, we investigated the synthetic conditions for microwave-assisted carboxymethylation of agar in this study and analyzed its influence on the physicochemical properties of the modified agar, comparing them to those of agar prepared using the conventional method. This study provides a technical reference for the development of a rapid and efficient preparation method for low-melting-point instant agar.

## 2. Results and Discussion

### 2.1. Effect of the Main Experimental Variables in the Microwave-Assisted Method on the DS of Carboxymethylated Agar

Carboxymethylation is the introduction of carboxymethyl groups in the chains of polysaccharides [14]. The hydroxyl groups in agar are successively substituted for alkoxide and carboxymethyl groups in an alkaline environment. The degree of substitution (DS) is used to evaluate the carboxymethylation yield because it has a significant effect on the physicochemical properties of modified agar and can be optimized by varying the experimental parameters. Optimization was performed using single-factor tests, and the influence of these factors on the DS is described in the subsequent sections.

#### 2.1.1. Microwave Power

**Figure 1:**
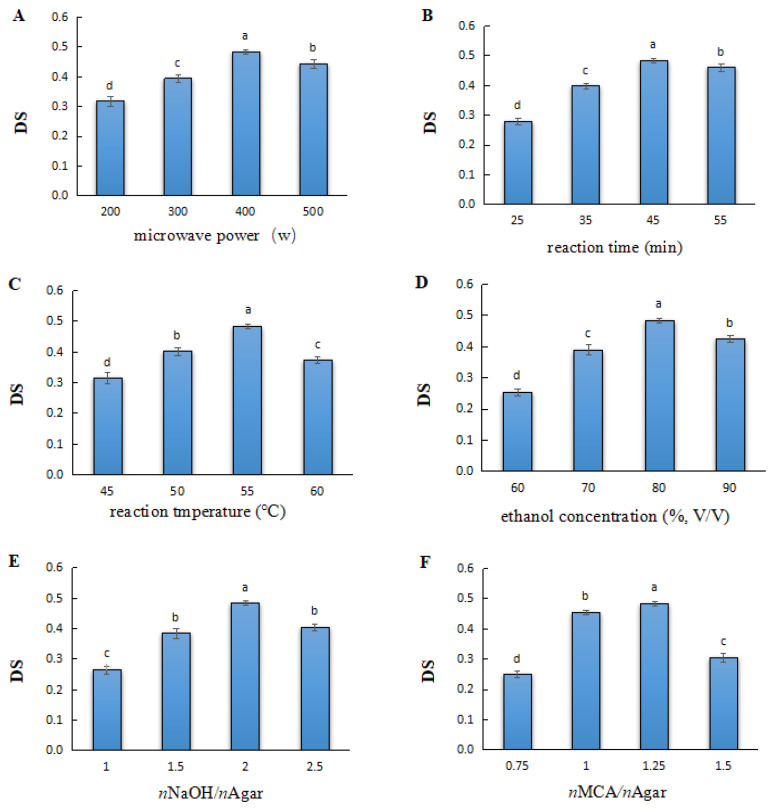
Effect of the main experimental variables in the microwave-assisted method on the DS of carboxymethyl agar: (**A**) microwave power, (**B**) reaction time, (**C**) reaction temperature, (**D**) ethanol concentration, (**E**) amounts of NaOH, and (**F**) monochloroacetic acid (MCA). Different letters above the bars in each histogram indicate significant differences (*p* < 0.05).

The microwave power was varied from 200 to 500 W (Figure 1A). With the increase in microwave power, the DS of the carboxymethyl agar first increased and then decreased. When the power was 400 W, the DS value reached the maximum (0.48). However, when the microwave power was increased to 500 W, the DS decreased to 0.43, indicating that the carboxymethyl yield could be improved by increasing the microwave power, but very high power microwaves were not conducive to the reaction. This could be due to the fact that a high microwave power causes the premature aggregation of polysaccharides, thus decreasing both contact with the reagents and carboxymethylation efficiency [19].

#### 2.1.2. Reaction Time

The reaction time was varied from 22 to 55 min (Figure 1B). With time, the DS increased in the first 45 min and then decreased, showing a continuous decrease from the maximum value obtained (0.47) at 45 min. Compared to conventional carboxymethyl synthesis methods that require hours [12], the microwave-assisted method was rapid and yielded samples having similar DS values.

#### 2.1.3. Reaction Temperature

The reaction temperature was varied from 45 to 60 °C (Figure 1C). Between 45 and 55 °C, the DS increased linearly with the increase in temperature. After peaking at 55 °C, the DS decreased, dropping to 0.37 at 60 °C. Therefore, the optimum temperature was 55 °C. Notably, a high temperature enhances the diffusion of reagents and increases polysaccharide swelling, thus increasing the reaction efficiency. However, overheating may degrade the agar molecules [15].

#### 2.1.4. Ethanol Concentration

The ethanol concentration was varied from 60% to 90% (volume fraction) (Figure 1D). With the increase in ethanol concentration, the DS first increased and then decreased. When the concentration of ethanol was increased to 80%, the DS reached its maximum value of 0.48. When the concentration was increased to 90%, the DS decreased to 0.45. Therefore, a higher concentration of ethanol is not always the best choice. In particular, a sufficient amount of water not only facilitates the dissolution of reagents in the reaction system, but also enhances the effects of the microwaves [20].

#### 2.1.5. Amount of NaOH

The molar NaOH-to-agar ratio was varied from 1.0 to 2.5 (Figure 1E). It was observed that the change in NaOH concentration in the reaction system affected the DS of the carboxymethyl agar to different degrees. When the ratio was increased from 1.0 to 2.0, the DS value increased. Next, after reaching a peak (0.47) at 2.0, the DS began to decrease, reaching 0.4 when the ratio reached 2.5. This trend resulted from the competition for MCA between NaOH and agar during carboxymethylation [21].

#### 2.1.6. Amount of MCA

The molar ratio of MCA and agar was varied from 0.75 to 1.50 (Figure 1F). The DS of carboxymethyl agar increased between the molar ratios of 0.75 and 1.25 and then decreased between those of 1.25 and 1.5, reaching a maximum of 0.47 when the MCA-to-agar molar ratio was 1.25. Notably, a higher MCA content led to the increased consumption of NaOH, which also acted as a swelling agent to facilitate the diffusion and penetration of the etherifying agent, and thus less amount of NaOH reacted with agar to improve the activity of the hydroxyl groups [22,23].

### 2.2. Effect of the Synthetic Method on the Solubility and Gel Properties of Carboxymethyl Agar Having Different DS Values

Carboxymethyl agar samples with different DS values were prepared using conventional and microwave-assisted methods. The solubility and gel properties of these samples are listed in Table 1.

Overall, the dissolution temperature, gel temperature, gel melting temperature, and gel strength of all carboxymethyl agar samples with different DS values prepared using the two synthetic methods were significantly lower than those of the unmodified agar (*p* < 0.05), and higher DS values resulted in a greater decrease in the gel strength. Therefore, carboxymethyl substitution changed the physical and chemical properties of agar to different degrees, and the degree of change was directly related to the DS values. Therefore, the selection of the appropriate carboxymethylation synthesis conditions can help achieve a DS tailored to the desired application.

Comparing the properties of the carboxymethyl agar prepared using different methods, we found that the dissolution temperature, gel temperature, and gel melting temperature of CAg were not significantly different from those of MAg (*p* > 0.05), whereas the gel strength of MAg was significantly higher than that of CAg (*p* < 0.05). The factors affecting the solubility and properties of agar mainly include its molecular weight and the ratio of hydrophilic groups to hydrophobic groups. When carboxymethyl groups are introduced, their strong hydrophilicity and steric hindrance effectively reduce the formation of intramolecular hydrogen bonds, improving the solubility of the agar. However, steric hindrance can also hinder the cross-linking of the agar chains, which is the reason for the decrease in gel strength [24]. In addition, the microwaves accelerated the rate of the synthetic reaction and shortened the reaction time; therefore, the agar remained under harsh reaction conditions for a shorter period, which prevented the degradation of the polysaccharide chains and possibly explains why the gel strength of MAg was higher than that of CAg [25].

### 2.3. Effect of the Synthetic Method on the Color and Transparency of Carboxymethyl Agars with Different DS Values

The yellowish color of unmodified agar reduces its applications as a food additive or substrate for microbial culture media. In particular, whiteness and gel transparency are important quality indicators for agar, and agar with high whiteness and transparency has many applications [26].

Comparing the color and transparency of the carboxymethyl agar to those of the original material (shown in Table 2), (1) the whiteness (*W*_H_) of all four carboxymethyl agar samples was significantly lower than that of the original agar (*p* < 0.05), and higher DS values resulted in lower whiteness for samples produced using the same method. However, there was no significant difference between the two preparation methods. (2) The b* (yellowness) values of carboxymethyl agar prepared using both methods increased significantly (*p* < 0.05), and, for the samples prepared using the same method, those with higher DS values had a more intense yellow color. Moreover, when the DS values of the samples were similar, the yellowness values of the samples prepared by the microwave method were higher than those of the samples prepared using the traditional method. (3) In contrast to the trend for whiteness, gel transparency (*T*) of carboxymethyl agar was greatly improved (*p* < 0.05) and was positively correlated with the DS. The microwave-assisted method had no significant effect on the transparency (*p* > 0.05) compared with the conventional method.

Therefore, in general, DS is the key factor affecting the whiteness and transparency of carboxymethyl agar, with the preparation method having little effect. Crucially, the hydrophilic interactions and steric hindrance of the carboxymethyl group make the modified agar molecules easier to stretch. After water infiltration, greater light transmission and lower reflection occurred; therefore, the gel obtained using carboxymethyl agar showed better transparency [10].

The highest transparency of MAg synthesized in this experiment was 94.1%, much higher than that of five types of commercial biochemical agar produced in our country and abroad, including Sigma A1296 (74.4%), Sigma A6686 (74.6%), Sigma V900500 (66.7%), Ourchem 1000058 (67.6%), and HKM 028990 (63.4%), and of a self-made carboxymethyl agar (84.5%), mentioned by Du et al. (2018) [27]. High-transparency agar can be widely used as a substrate for microorganism cultures with higher transparency requirements, which enables the earlier detection of growing colonies [27,28], and as an agar-based edible film for food packaging that does not affect food appearance [29]. It also can be used to prepare agar-based hydrogels or mixed hydrogels as thickeners, stabilizers, and gelling agents [30].

### 2.4. Effect of the Synthetic Method on the Textural Properties of Carboxymethyl Agar with Different DS

Next, the gel texture characteristics of the modified agar prepared using the two methods were analyzed, including hardness, cohesiveness, springiness, and chewiness. As shown in Table 3, the cohesiveness and springiness of all carboxymethyl agar gels were significantly improved compared to those of the original agar gel (*p* < 0.05), whereas the hardness and chewiness of the modified agar gel decreased significantly (*p* < 0.05), which was similar to the change in gel strength. In particular, for samples prepared using the same method, a higher DS value resulted in greater changes in strength. The decrease in hardness and chewiness indicated that the ability of the carboxymethyl agar gel to resist external forces was weakened, whereas the increase in cohesiveness and springiness suggested that the carboxymethyl agar gel became soft and difficult to break, and its flexibility was improved, which is related to the gel network structure and water content [31].

For samples with similar DS values, the microwave-assisted method had greater effects on the tested textural properties than the conventional method, except for chewiness. However, higher DS values resulted in smaller changes.

### 2.5. Effect of the Synthetic Method on the Molecular Structure of Carboxymethyl Agar with Different DS Values

**Figure 2:**
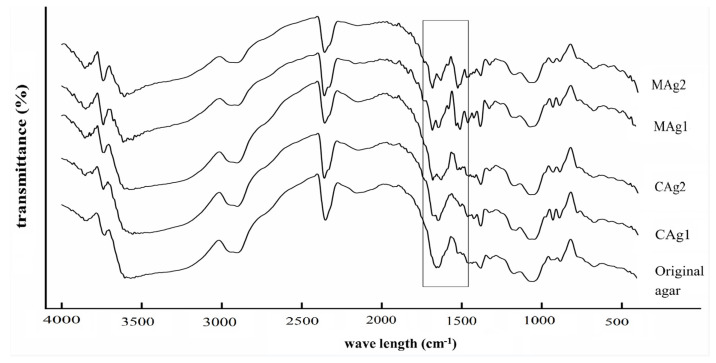
FT-IR spectra of unmodified and carboxymethyl agar. CAg1 and CAg2, carboxymethyl agar prepared using the conventional method, with DS of 0.25 and 0.47, respectively; MAg1 and MAg2, carboxymethyl agar prepared using the microwave-assisted method, with DS values of 0.25 and 0.48, respectively.

The changes in the molecular structure of the agar after carboxymethylation as determined by FT-IR measurements are shown in Figure 2.

The spectrum of unmodified agar presents a wide band at 3700–3000 cm^−1^, which corresponds to the hydroxyl (–OH) groups in the agar chains. The absorption band near 2900 cm^−1^ corresponds to the symmetric stretching of –CH_2_ groups, which is characteristic of polysaccharides [32].

After carboxymethylation, the intensity of the absorption band at 3700–3000 cm^−1^ decreased compared to that of the unmodified agar. This indicates that the number of hydroxyl groups in the polysaccharide chains decreased, possibly as a result of substitution by carboxymethyl groups. Meanwhile, a new band consistent with the asymmetric stretching of –C=O groups appeared at 1640 cm^−1^, which corresponded to the carboxylate anion (–COO^−^). In addition, a weak absorption band corresponding to the stretching of –CH groups appeared near 1439 cm^−1^. These results indicate that the carboxymethyl groups were successfully introduced into the agar chains [14].

As shown in Figure 2, with the increase in the DS, the intensity of the band corresponding to the –OH groups decreased, whereas the absorption band corresponding to the –COO^−^ groups at 1640 cm^−1^ increased gradually, indicating the addition of carboxymethyl groups and suggesting that the changes in the physical and chemical properties of the agar were closely related to the introduction of these groups [19].

The FT-IR spectra of samples with the same DS prepared using both conventional and microwave methods were the same, indicating that the thermal and non-thermal effects of the microwaves did not change the molecular structure of the agar polysaccharides nor produced new functional groups, but instead only accelerated the chemical reaction.

### 2.6. Effect of the Synthetic Method on the Zeta Potential and Particle Size of Carboxymethyl Agar with Different DS Values

**Figure 3:**
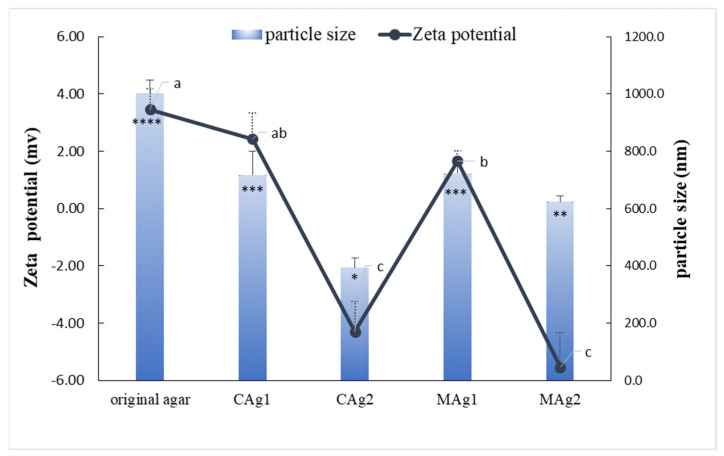
Effect of the synthetic method on zeta potential and particle size of unmodified and modified agar. Note: Different letters on the line and different number of symbols (*) above the columns indicate significant differences (*p* < 0.05).

In this study, zeta potential measurements were used to characterize the interactions between the agar molecules, the charge density on the particle surface, and the stability of the suspension; the results are shown in Figure 3. The zeta potential of the original agar was positive, but the zeta potential of the carboxymethylated agar decreased and gradually became negative with the increase in DS because of the introduction of the negatively charged groups. When the DS was 0.47 or 0.48, the absolute value of the zeta potential of the modified agar was greater than that of the original agar with a DS of 0.25. This showed that the higher DS resulted in a more negative charge and a lower zeta potential. In general, the higher the surface charge of colloidal particles, the greater the repulsion, the less likely colloidal particles are to aggregate, and the more stable the colloidal system is. Specifically, the introduction of more carboxymethyl groups resulted in a lower zeta potential and a stronger electrostatic repulsion [33]. Therefore, the carboxymethyl agar gel with a higher DS was more stable. Furthermore, for samples with the same DS, the different preparation methods had no significant effect on the zeta potential (*p* < 0.05).

Generally, larger particles are unstable and more likely to aggregate. Particle size analysis (Figure 3) showed that the unmodified agar particles were larger than the modified agar particles, suggesting that the aggregation of the former in aqueous solution was stronger than that of the latter. With the increase in DS, the particle size of the carboxymethyl agar decreased significantly (*p* < 0.05). This could be because the electrostatic repulsion between the agar particles with a high DS reduced their aggregation in aqueous solution, leading to a decrease in particle size. For samples having the same DS value, the two preparation methods had no significant effect on the particle size in aqueous solution (*p* < 0.05), which is consistent with the zeta potential results.

### 2.7. Effect of the Synthetic Method on the Molecular Weight of Carboxymethyl Agar with Different DS Values

Several important properties of polysaccharides are closely related to their molecular weight and its distribution, but extremely high molecular weights can have a negatively effect on polysaccharide processability. Therefore, considering applicability and processability, the molecular weight of polysaccharides, such as agar, must be controlled within an appropriate range. The molecular weight is the key factor affecting the solubility and gel properties of agar, and the trends in these properties are contradictory. In other words, decreasing the solubility temperature requires compromising the gel strength [1,6].

The results of the molecular weight analysis of the original and carboxymethyl agar are shown in Table 4 and Figure S1–S6. The weight-average molecular weight (*M*_w_) of the original agar was approximately 4.5 × 10^4^ g·mol^−1^, and the polydispersity index (*M*_w_/*M*_n_, where *M*_n_ is the number-average molecular weight) was 2.593, indicating that the molecular weights were widely dispersed. After carboxymethylation, both *M*_w_ and *M*_n_ of the modified agar decreased sharply, and higher values of DS resulted in decreased *M*_w_/*M*_n_ values, indicating that carboxymethylation significantly degraded the agar.

For samples with a similar DS, the molecular weight of the carboxymethyl agar prepared using the microwave-assisted method was much higher than that of the agar obtained with the conventional method, suggesting that the microwave-assisted method effectively protected the agar from degradation, This could be due to the fact that the microwave treatment accelerated the reaction rate, allowing the carboxymethylation reaction to complete rapidly and separating the agar from the reaction system, thus its preventing excessive degradation.

### 2.8. Effect of the Synthetic Method on the Surface Morphology of Carboxymethyl Agar Having Different DS Values

**Figure 4:**
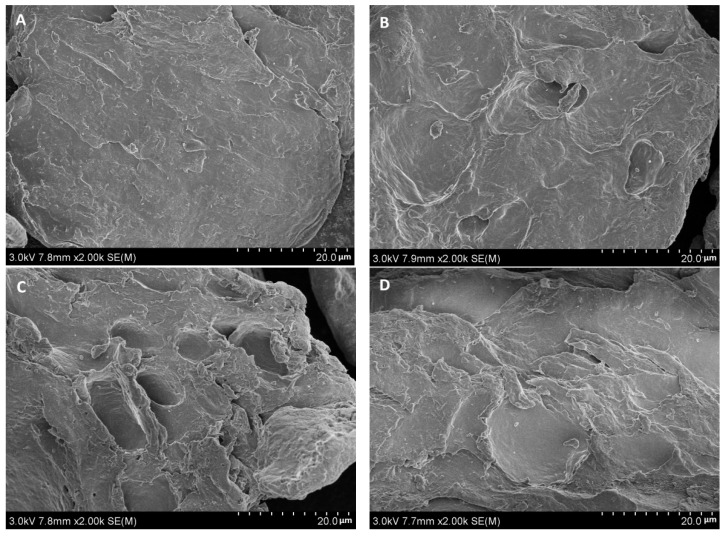

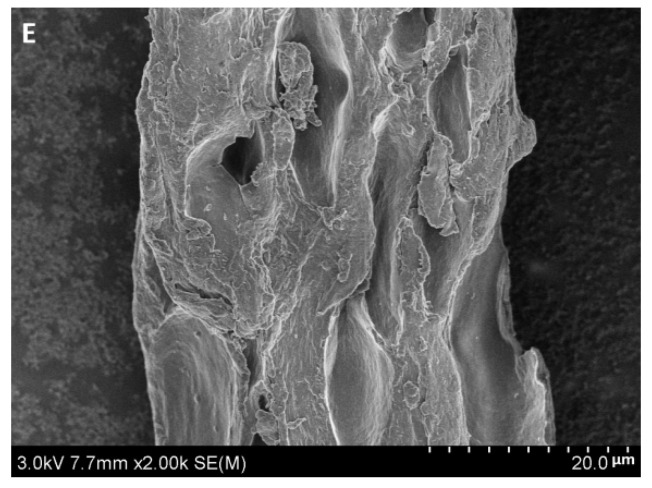
Effect of the synthetic method on the microstructure of carboxymethyl agar: (**A**) original agar, (**B**) CAg1, (**C**) CAg2, (**D**) MAg1, and (**E**) MAg2.

The microstructures of the original and carboxymethyl agar samples with different DS values were analyzed by scanning electron microscopy, as shown in Figure 4. The surface of the original agar (Figure 4A) was relatively smooth, without apparent holes and cracks, and the particle size was relatively large. In contrast, the particle surface of the carboxymethyl agar (Figure 4B–E) was rough and covered with depressions. For samples prepared using the microwave method, higher DS values resulted in a larger area covered with holes and depressions. However, for samples having the same DS value, the surface structures of the samples prepared by both methods were similar. Therefore, the microstructure of the carboxymethyl agar was greatly affected by the DS itself rather than by the preparation method. The origin of these morphological differences could be electrostatic repulsion between carboxymethyl groups and the destructive effect of the alkaline reaction system [34].

## 3. Conclusions

The modification of agar through carboxymethylation improved its physical and chemical properties, and the degree of change in the properties differed compared to the preparation method. However, the DS was the key factor affecting the physicochemical properties of the modified agar, but the concentration and ratio of reagents involved in the reaction, the reaction temperature and time, and the microwave power also had an effect. Of the physicochemical properties affected by carboxymethylation, cohesiveness, springiness, and transparency increased significantly, whereas dissolution temperature, gel temperature, gel melting temperature, gel strength, whiteness, hardness, chewiness, molecular zeta potential, particle size, and molecular weight decreased significantly. In addition, the surface morphology of the modified agar particles showed more depressions and holes than that of the unmodified agar, and the samples having higher DS values showed more significant changes. Therefore, concerning practical applications, modification should be carried out to obtain DS values suitable for the final application.

Compared to the conventional synthetic method, the effects of the microwave-assisted method on the physicochemical properties of the modified agar were as follows: (1) the microwave-assisted method greatly decreased the time required for carboxymethylation and significantly improved the reaction efficiency. Considering the preparation of carboxymethyl agar with DS values of 0.25 and 0.48 as examples, the microwave-assisted method required only 25 and 45 min, respectively, whereas the conventional method took 45 and 240 min, respectively. This difference is very significant. (2) The microwave-assisted method effectively protected the agar molecules from degradation; thus, the molecular weight of the modified agar prepared using this method was greater than that of agar obtained with the conventional method. (3) The effect of the microwave-assisted method on hardness, cohesiveness, and springiness of the agar gel was more significant than that of the conventional method, whereas the impact of the microwave method on gel strength was significantly lower than that of the conventional method. There were no significant differences in other physical and chemical properties.

## 4. Materials and Methods

### 4.1. Materials

Food-grade agar was purchased from Chenghai Agar Factory (Shantou, China). Sodium hydroxide (NaOH), monochloroacetic acid (MCA), and absolute ethanol were purchased from Guangzhou Chemical Reagent Factory (Guangzhou, China), and all reagents were of analytical grade.

### 4.2. Single-Factor Tests of the Microwave-Assisted Synthesis of Carboxymethyl Agar

The effects of the microwave power, reaction temperature, reaction time, concentration of ethanol, and amounts of NaOH and MCA on the microwave-assisted synthesis of carboxymethyl agar were analyzed with the single-factor test method, using the substitution degree (DS) as the evaluation index. The settings for each factor are listed in Table 5.

### 4.3. Synthesis of Carboxymethyl Agar with Different DS Values Using Microwave-Assisted and Conventional Methods

Agar powder (5 g) was dispersed in ethanol (50 mL, 80% volume fraction) and stirred for 0.5 h in a conical flask to achieve full swelling. The flask was equipped with a temperature controller and a mechanical stirrer. Then, NaOH was added to the conical flask for alkalization at 40 °C for 2 h (for the conventional method) or at 45 °C for 20 min (for the microwave-assisted method that was carried out in a MAS II microwave-assisted synthesizer (Shanghai, China); the mixture was heated by microwaves from this step). Subsequently, MCA and NaOH were added sequentially in the following molar ratios: *n*NaOH/*n*Agar (2.0) and *n*MCA/*n*Agar (1.00). Subsequently, the reaction temperature was increased to 55 °C and maintained for the corresponding time (see Table 6). After completion, the reaction solution was allowed to cool to 20 °C, neutralized with 1 mol·L^−1^ glacial acetic acid, and filtered using vacuum filtration. The residue was washed repeatedly with 60% ethanol until no chloride ions (Cl^−^) were detected in the filtrate using silver nitrate, dried in an oven at 60 °C, and then finally crushed to 80 mesh. The carboxymethyl agar sample prepared using the conventional method was denoted CAg, whereas the sample prepared using the microwave-assisted method was denoted MAg.

### 4.4. Determination of DS

The DS of the samples was determined using a neutralization titration method [14]. Carboxymethyl agar (0.1 g) was dissolved in 25 mL of distilled water, and then 25 mL of NaOH standard solution (0.1 mol·L^−1^) was added to the conical flask. After stirring for 10–15 min, the mixture was titrated with a 0.1 mol·L^−1^ HCl standard solution until it became colorless, and phenolphthalein was used as an indicator. The titration was repeated three times, and the average volume of HCl was used for the calculations. The DS was calculated using Equations (1) and (2).

(1) $$\mathrm{DS}=\frac{0.306X}{1-0.058X}$$

(2) $$X=\frac{V_{0}M_{0}-V_{1}M_{1}}{W}$$

Here, 306 is the molar mass of the disaccharide unit (g·mol^−1^) [11], 58 is the molar mass of the introduced carboxymethyl group (g·mol^−1^), *V*_0_ is the volume of NaOH added (mL), *M*_0_ is the concentration of NaOH added (mol·L^−1^), *V*_1_ is the consumed volume of HCl (mL), *M*_1_ is the concentration of HCl (mol·L^−1^), and *W* is the sample weight (g).

### 4.5. Determination of the Physicochemical Properties of Carboxymethyl Agar

The dissolving temperature, gelling temperature, and gel melting temperature were analyzed using the method described by Cao et al. [12]. The gel strength was determined using the method described by Lee et al. [35].

The color, as given in CIE coordinates (L*, a*, and b*), was determined according to the method described by An et al. [26] with a CR-400 colorimeter, and the Hunter whiteness index (*W*_H_) was calculated using Equation (3).

(3) $$W_{H}=100-\sqrt{{(100-L^{*})}^{2}+a^{*}{}^{2}+b^{*}{}^{2}}$$

Here, *W*_H_ is the Hunter whiteness value, L* is the lightness–darkness value, a* is the red–green value, and b* is the yellow–blue value.

The transparency was determined using the method described by Xiao et al. [36]. Briefly, a sample of the hot solution was placed in a cuvette and stored at 20 °C for 12 h. Then, the transparency was measured using transmittance (%, *T*) at 700 nm, with distilled water as the reference sample.

The textural properties of the agar gel, including hardness, cohesiveness, springiness, and chewiness, were tested using a CT3 texture analyzer (Brookfield, Middleboro, USA). The sample gel was cut into cylindrical samples with a diameter of 15 mm and a height of 15 mm, and the analysis was carried out in texture profile analysis (TPA) mode using a 10 mm flattened cylindrical probe, a constant speed of 1 mm·s^−1^, and a target 30% compression ratio.

All the measurements were performed in triplicate on a 1.5% (weight volume percentage) sample solution.

### 4.6. Fourier Transform Infrared (FT-IR) Spectroscopy

The FT-IR spectra of the unmodified and modified agar samples were obtained using the method described by Gope et al. [37] with an IRAffinity-1 FT-IR spectrometer (Shimadzu, Kyoto, Japan).

### 4.7. Determination of the Zeta Potential and Particle Size

The zeta potential and particle size were determined using a Malvern Zetamaster instrument (Malvern Instruments Ltd., Malvern, UK). Carboxymethyl agar was prepared in a 0.5 mg·mL^−1^ solution and analyzed using the method described by Crudden et al. [38]. The zeta potential and particle size of each sample were determined in triplicate.

### 4.8. Molecular Weight Determination

The average molecular weight of the agar samples was estimated using gel permeation chromatography (GPC, Agilent, Palo Alto, USA) as described by Sugumaran et al. [39]. The chromatograph was equipped with a PLgel column having 5 μm pores and a refractive index (RI) detector. The sample was prepared as a solution with a concentration of 2.0 mg·mL^−1^ and filtered through a 0.45 μm microporous cellulose acetate membrane. The injection volume was 20 μL.

### 4.9. Morphological Structure

The morphological structure of the samples was evaluated based on the method described by Gope et al. [37] using an SU8020 scanning electron microscope (Hitachi Ltd., Tokyo, Japan). A small amount of carboxymethyl agar sample was first adhered to a double-sided adhesive tape, and the tapes were examined under a microscope at an accelerating potential of 3 kV at 2000× magnification.

### 4.10. Statistical Analysis

Data were subjected to the analysis of variance (ANOVA), and the comparison of means was performed using Tukey’s honestly significant difference (HSD) test. Differences were considered significant at *p* < 0.05. Statistical computations and analyses were conducted using SPSS (version 24.0).

## Figures and Tables

**Table 1:** Effect of the synthetic method on the physicochemical properties of carboxymethyl agar.

Samples	Dissolving Temperature (°C)	Gelling Temperature (°C)	Gel Melting Temperature (°C)	Gel Strength (g cm^−2^)
Original agar	97.6 ± 1.3 ^a^	38.7 ± 0.7 ^a^	94.7 ± 0.8 ^a^	1145 ± 28 ^a^
CAg1	77.3 ± 1.8 ^b^	36.4 ± 0.5 ^b^	68.2 ± 0.4 ^b^	446 ± 23 ^c^
CAg2	64.5 ± 0.9 ^c^	30.7 ± 0.5 ^c^	59.6 ± 0.5 ^d^	128 ± 8 ^e^
MAg1	78.3 ± 1.1 ^b^	37.1 ± 0.4 ^b^	69.5 ± 0.7 ^b^	545 ± 19 ^b^
MAg2	65.7 ± 0.6 ^c^	31.3 ± 0.7 ^c^	60.3 ± 1.1 ^d^	152 ± 5 ^d^

Note: CAg1 and CAg2, carboxymethyl agar prepared using the conventional method, with DS of 0.25 and 0.47, respectively; MAg1 and MAg2, carboxymethyl agar prepared using the microwave-assisted method, with DS of 0.25 and 0.48, respectively. Different letters in the same column indicate significant differences (*p* < 0.05).

**Table 2:** Effect of the synthetic method on color and gel transparency of carboxymethyl agar.

Samples	L*	a*	b*	*W*_H_	*T* (%)
Original agar	87.98 ± 0.23 ^a^	0.72 ± 0.03 ^e^	16.45 ± 0.11 ^e^	79.61 ± 0.7 ^a^	35.6 ± 0.9 ^c^
CAg1	86.23 ± 0.42 ^b^	1.65 ± 0.06 ^c^	17.63 ± 0.23 ^d^	77.56 ± 0.9 ^b^	82.1 ± 1.4 ^b^
CAg2	84.11 ± 0.33 ^c^	2.71 ± 0.08 ^a^	19.16 ± 0.14 ^b^	74.96 ± 0.4 ^c^	93.3 ± 1.9 ^a^
MAg1	86.68 ± 0.16 ^b^	1.48 ± 0.10 ^d^	17.95 ± 0.07 ^c^	77.60 ± 0.4 ^b^	81.5 ± 0.5 ^b^
MAg2	84.65 ± 0.21 ^c^	2.53 ± 0.06 ^b^	19.68 ± 0.17 ^a^	74.91 ± 0.6 ^c^	94.1 ± 1.3 ^a^

Note: L*, the lightness–darkness value; a*, the red–green value; b*, the yellow–blue value; *W*_H_, the Hunter whiteness value; *T*, transparency. Different letters in the same column indicate significant differences (*p* < 0.05).

**Table 3:** Effect of the synthetic method on the textural properties of carboxymethyl agar.

Samples	Hardness (g)	Cohesiveness	Springiness (mm)	Chewiness (mJ)
Original agar	1532.67 ± 34.05 ^a^	0.59 ± 0.05 ^d^	3.81 ± 0.08 ^c^	34.94 ± 1.87 ^a^
CAg1	1143.25 ± 55.51 ^b^	0.69 ± 0.02 ^c^	4.11 ± 0.11 ^b^	31.77 ± 3.39 ^c^
CAg2	282.50 ± 10.21 ^d^	0.67 ± 0.08 ^c^	4.32 ± 0.07 ^a^	9.73 ± 0.25 ^d^
MAg1	1000.67 ± 23.03 ^c^	0.80 ± 0.05 ^b^	4.32 ± 0.08 ^a^	32.46 ± 4.48 ^b^
MAg2	263.83 ± 33.13 ^d^	0.89 ± 0.01 ^a^	4.35 ± 0.1 ^a^	9.99 ± 1.27 ^d^

Different letters in the same column indicate significant differences (*p* < 0.05).

**Table 4:** Effect of the synthetic method on the molecular weight of carboxymethyl agar samples having different DS values.

Samples	*M*_w_ (g·mol^−1^)	*M*_n_ (g·mol^−1^)	*M*_w_/*M*_n_
Original agar	4.498 × 10^4^	1.735 × 10^4^	2.593
CAg1	9.561 × 10^3^	5.837 × 10^3^	1.638
CAg2	2.506 × 10^3^	2.104 × 10^3^	1.191
MAg1	1.542 × 10^4^	1.236 × 10^4^	1.248
MAg2	7.463 × 10^3^	5.193 × 10^3^	1.437

**Table 5:** Single-factor tests of the microwave-assisted synthesis of carboxymethyl agar.

No.	Microwave Power (W)	Temperature (°C)	Time (min)	Ethanol Concentration (%)	*n*NaOH/*n*Agar	*n*MCA/*n*Agar
1	500	55	45	80	2.0	1.00
2	400	55	45	80	2.0	1.00
3	300	55	45	80	2.0	1.00
4	200	55	45	80	2.0	1.00
5	400	60	45	80	2.0	1.00
6	400	50	45	80	2.0	1.00
7	400	45	45	80	2.0	1.00
8	400	55	55	80	2.0	1.00
9	400	55	35	80	2.0	1.00
10	400	55	25	80	2.0	1.00
11	400	55	45	60	2.0	1.00
12	400	55	45	70	2.0	1.00
13	400	55	45	90	2.0	1.00
14	400	55	45	80	2.5	1.00
15	400	55	45	80	1.5	1.00
16	400	55	45	80	1.0	1.00
17	400	55	45	80	2.0	1.5
18	400	55	45	80	2.0	1.25
19	400	55	45	80	2.0	0.75

Note: *n*NaOH/*n*Agar is the molar ratio of NaOH to agar, and *n*MCA/*n*Agar is the molar MCA-to-agar ratio. Both are with respect to agarbiose.

**Table 6:** Synthetic conditions for carboxymethyl agar with similar DS values.

Sample	Microwave Power (W)	Time (min)	Temperature (°C)	DS
CAg1	−	45	55	0.25
CAg2	−	240	55	0.47
MAg1	400	25	55	0.25
MAg2	400	45	55	0.48

## Data Availability

The data presented in this study are available on request from the corresponding author.

## Supplementary Materials

The following supporting information can be downloaded at: https://www.mdpi.com/article/10.3390/gels8030162/s1, Figure S1: GPC chromatogram of original agar; Figure S2: GPC chromatogram of carboxymethyl agar prepared using conventional method with DS of 0.25 (CAg1); Figure S3: GPC chromatogram of carboxymethyl agar prepared using conventional method with DS of 0.47 (CAg2); Figure S4: GPC chromatogram of carboxymethyl agar prepared using microwave-assisted method with DS of 0.25 (MAg1); Figure S5: GPC chromatogram of carboxymethyl agar prepared using microwave-assisted method with DS of 0.48 (MAg2); Figure S6: GPC chromatogram of dextran standards.

## References

[1] Abdul Khalil H.P.S., Lai T.K., Tye Y.Y., Rizal S., Chong E.W.N., Yap S.W., Hamzah A.A., Nurul Fazita M.R., Paridah M.T. (2018). A review of extractions of seaweed hydrocolloids: Properties and applications. Express Polym. Lett..

[2] Armisén R. (1991). Agar and agarose biotechnological applications. Hydrobiologia.

[3] Zarrintaj P., Manouchehri S., Ahmadi Z., Saeb M.R., Urbanska A.M., Kaplan D.L., Mozafari M. (2018). Agarose-based biomaterials for tissue engineering. Carbohydr. Polym..

[4] Wang L.J., Shen Z.P., Mu H.M., Lin Y., Zhang J.L., Jiang X.L. (2017). Impact of alkali pretreatment on yield, physico-chemical and gelling properties of high quality agar from Gracilaria tenuistipitata. Food Hydrocoll..

[5] Zhang N., Wang J.L., Ye J., Zhao P., Xiao M.T. (2018). Oxyalkylation modification as a promising method for preparing low-melting-point agarose. Int. J. Biol. Macromol..

[6] Bertasa M., Dodero A., Alloisio M., Vicini S., Riedo C., Sansonetti A., Scalarone D., Castellano M. (2020). Agar gel strength: A correlation study between chemical composition and rheological properties. Eur. Polym. J..

[7] Xia K., Liu X., Zhao J.K., Zhang X.D. (2014). The physicochemical property characterization of agar acetate. Carbohydr. Polym..

[8] Belay M., Tyeb S., Rathore K., Kumar M., Verma V. (2020). Synergistic effect of bacterial cellulose reinforcement and succinic acid crosslinking on the properties of agar. Int. J. Biol. Macromol..

[9] Zhang C., An D., Xiao Q., Weng H.F., Zhang Y.H., Yang Q.M., Xiao A. (2020). Preparation, characterization, and modification mechanism of agar treated with hydrogen peroxide at different temperatures. Food Hydrocoll..

[10] Xiao Q., An D., Zhang C., Weng H.F., Zhang Y.H., Chen F.Q., Xiao A. (2020). Agar quality promotion prepared by desulfation with hydrogen peroxide. Int. J. Biol. Macromol..

[11] Xiao Q., Weng H.F., Chen G., Xiao A.F. (2019). Preparation and characterization of octenyl succinic anhydride modified agarose derivative. Food Chem..

[12] Cao M.Z., Liu X., Luan J.M., Zhang X.D. (2014). Characterization of physicochemical properties of carboxymethyl agar. Carbohydr. Polym..

[13] Wang L.N., Yang S.L., Qi B., Yang X.Q., Li C.S., Ma H.X., Hu X. (2021). Effect of three modification methods on physicochemical properties of agar. S. China Fish. Sci..

[14] Chakka V.P., Zhou T. (2020). Carboxymethylation of polysaccharides: Synthesis and bioactivities. Int. J. Biol. Macromol..

[15] Singh A.V., Nath L.K., Guha M., Kumar R. (2011). Microwave assisted synthesis and evaluation of cross-linked carboxymethylated sago starch as superdisintegrant. Pharmacol. Pharm..

[16] Kumar Y., Singh L., Sharanagat V.S., Patel A., Kumar K. (2020). Effect of microwave treatment (low power and varying time) on potato starch: Microstructure, thermo-functional, pasting and rheological properties. Int. J. Biol. Macromol..

[17] Kaplan Ö., Tosun N.G., Özgür A., Tayhan S.E., Bilgin S., Türkekul İ., Gökce İ. (2021). Microwave-assisted green synthesis of silver nanoparticles using crude extracts of *Boletus edulis* and *Coriolus versicolor*: Characterization, anticancer, antimicrobial and wound healing activities. J. Drug Deliv. Sci. Technol..

[18] Hou C.M., Chen Y.F., Chen W.N., Li W. (2011). Microwave-assisted methylation of cassava starch with dimethyl carbonate. Carbohydr. Res..

[19] Liu J., Ming J., Li W.J., Zhao G.H. (2012). Synthesis, characterisation and in vitro digestibility of carboxymethyl potato starch rapidly prepared with microwave-assistance. Food Chem..

[20] Balsamo V., López-Carrasquero F., Laredo E., Conto K., Contreras J., Feijoo J.L. (2011). Preparation and thermal stability of carboxymethyl starch/quaternary ammonium salts complexes. Carbohydr. Polym..

[21] Silva D.A., de Paula R.C.M., Feitosa J.P.A., de Brito A.C.F., Maciel J.S., Paula H.C.B. (2004). Carboxymethylation of cashew tree exudate polysaccharide. Carbohydr. Polym..

[22] Fan L.H., Wang L.B., Gao S., Wu P.H., Li M.J., Xie W.G., Liu S.H., Wang W. (2011). Synthesis, characterization and properties of carboxymethyl kappa carrageenan. Carbohydr. Polym..

[23] Milotskyi R., Bliard C., Tusseau D., Benoit C. (2018). Starch carboxymethylation by Reactive Extrusion: Reaction kinetics and Structure Analysis. Carbohydr. Polym..

[24] Lawal O.S., Lechner M.D., Kulicke W.M. (2008). Single and multi-step carboxymethylation of water yam (*Dioscorea alata*) starch: Synthesis and characterization. Int. J. Biol. Macromol..

[25] Staroszczyk H., Fiedorowicz M., Opalińska-Piskorz J., Tylingo R. (2013). Rheology of potato starch chemically modified with microwave-assisted reactions. LWT–Food Sci. Technol..

[26] An D., Xiao Q., Zhang C., Cai M.H., Zhang Y.H., Weng H.F., Chen F.Q., Xiao A. (2021). Preparation, characterization, and application of high-whiteness agar bleached with hydrogen peroxide. Food Hydrocoll..

[27] Du J.H., Li T., Ni H., Jiang Z.D., Xiao A.F., Zhu Y.B. (2018). Preparation of Biochemical Agar Using Gracilaria Agar as Raw Material (in Chinese). Chin. J. Process Eng..

[28] Kim H., Ryoo S.W. (2011). Exploitation of Culture Medium for Mycobacterium tuberculosis. J. Bacteriol. Virol..

[29] Mostafavi F.S., Zaeim D. (2020). Agar-based edible films for food packaging applications—A review. Int. J. Biol. Macromol..

[30] Zhang Z., Wang X., Wang Y., Hao J. (2018). Rapid-forming and self-healing agarose-based hydrogels for tissue adhesives and potential wound dressings. Biomacromolecules.

[31] Soto-Reyes N., Temis-Pérez A.L., López-Malo A., Rojas-Laguna R., Sosa-Morales M.E. (2015). Effects of shape and size of agar gels on heating uniformity during pulsed microwave treatment. J. Food Sci..

[32] Singh V., Maurya S. (2010). Microwave synthesis, characterization, and zinc uptake studies of starch-graft-poly (*ethyl acrylate*). Int. J. Biol. Macromol..

[33] Liu Y., Lu K.Y., Hu X.T., Jin Z.Y., Miao M. (2020). Structure, properties and potential applications of phytoglycogen and waxy starch subjected to carboxymethylation. Carbohydr. Polym..

[34] Wang L.F., Pan S.Y., Hu H., Miao W.H., Xu X.Y. (2010). Synthesis and properties of carboxymethyl kudzu root starch. Carbohydr. Polym..

[35] Lee W.K., Namasivayam P., Ho C. (2014). Effects of sulfate starvation on agar polysaccharides of Gracilaria species (*Gracilariaceae, Rhodophyta*) from Morib, Malaysia. J. Appl. Phycol..

[36] Xiao Q., Weng H.F., Ni H., Hong Q.L., Lin K.H., Xiao A.F. (2019). Physicochemical and gel properties of agar extracted by enzyme and enzyme-assisted methods. Food Hydrocoll..

[37] Gope S., Samyor D., Paul A.K., Das A.B. (2016). Effect of alcohol-acid modification on physicochemical, rheological and morphological properties of glutinous rice starch. Int. J. Biol. Macromol..

[38] Crudden A., Afoufa-Bastien D., Fox P.F., Brisson G., Kelly A.L. (2005). Effect of hydrolysis of casein by plasmin on the heat stability of milk. Int. Dairy J..

[39] Sugumaran K.R., Sindhu R.V., Sukanya S., Aiswarya N., Ponnusami V. (2013). Statistical studies on high molecular weight pullulan production in solid state fermentation using jack fruit seed. Carbohydr. Polym..

