# Supplementary material for: Comparison of the Physicochemical Properties of Carboxymethyl Agar Synthesized by Microwave-Assisted and Conventional Methods
Source: Gels. 2022 Mar 4;8(3):162. doi: 10.3390/gels8030162 (PMC8951826; doi:10.3390/gels8030162)
Supplement: Supplementary file 1 [file gels-08-00162-s001.zip › gels-1596005-supplementary.pdf]

SUPPLEMENTARY INFORMATION

Comparison of the Physicochemical Properties of Carboxymethyl Agar Synthesized by Microwave-Assisted and Conventional Methods

Bo Qi [1,2], Shaoling Yang [1,3,*], Yongqiang Zhao [1,*], Yueqi Wang [1], Xianqing Yang [1], Shengjun Chen [1], Yanyan Wu [1], Chuang Pan [1], Xiao Hu [1], Chunsheng Li [1] and Lunan Wang [1,4]

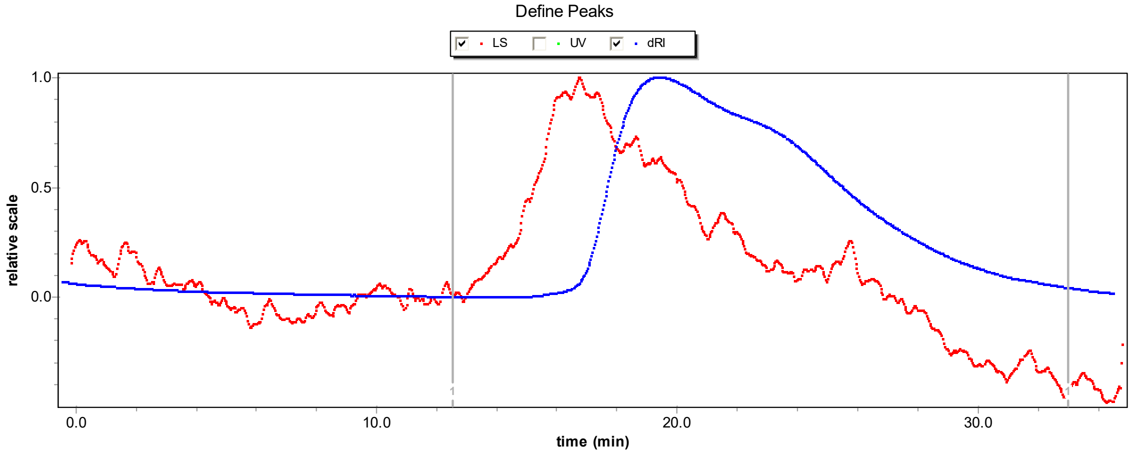

FigureS1. GPC chromatogram of original agar

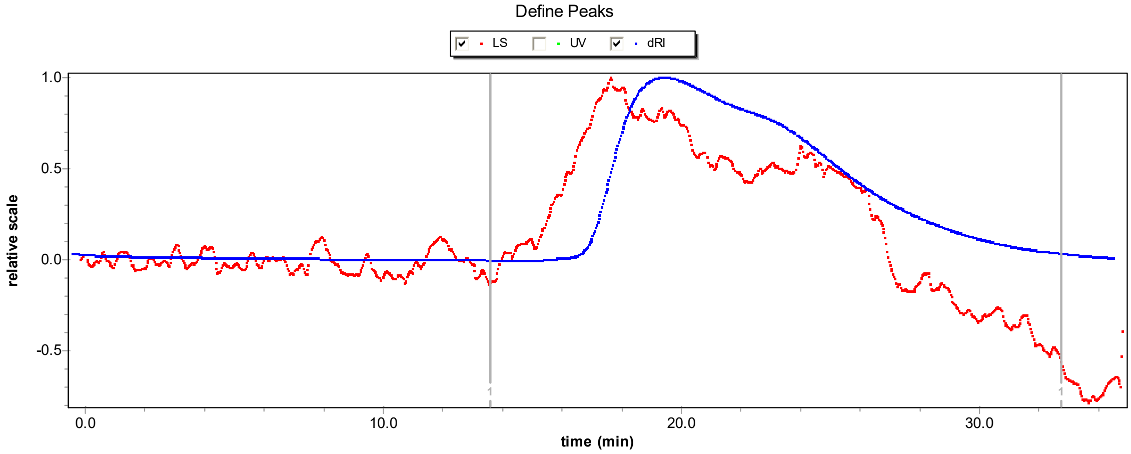

Figure S2. GPC chromatogram of carboxymethyl agar prepared using conventional method with DS of 0.25 (CAg1)

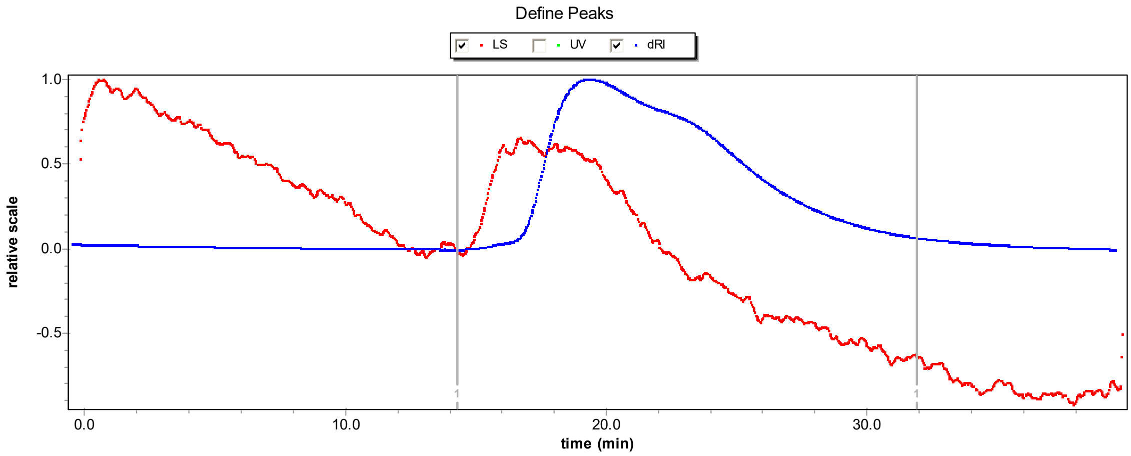

Figure S3. GPC chromatogram of carboxymethyl agar prepared using conventional method with DS of 0.47 (CAg2)

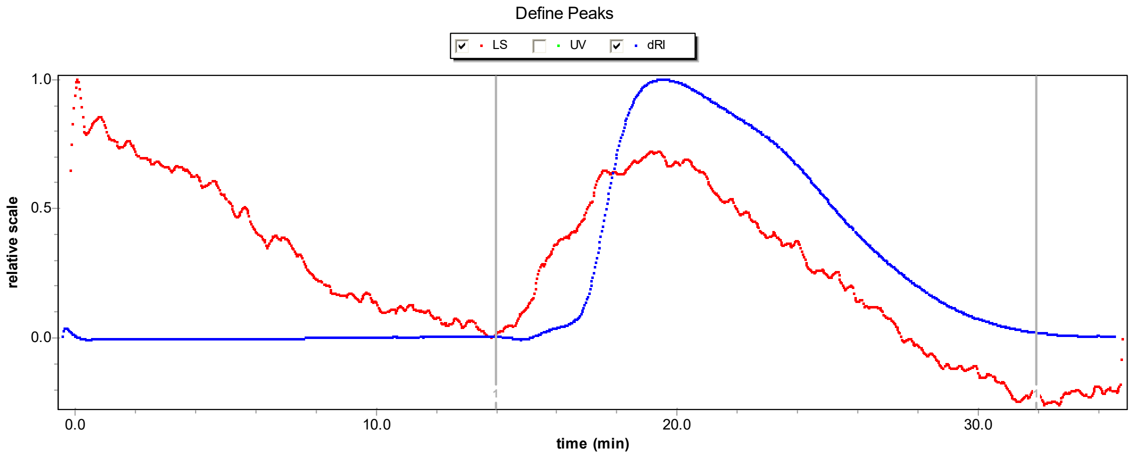

Figure S4. GPC chromatogram of carboxymethyl agar prepared using microwave-assisted method with DS of 0.25 (MAg1)

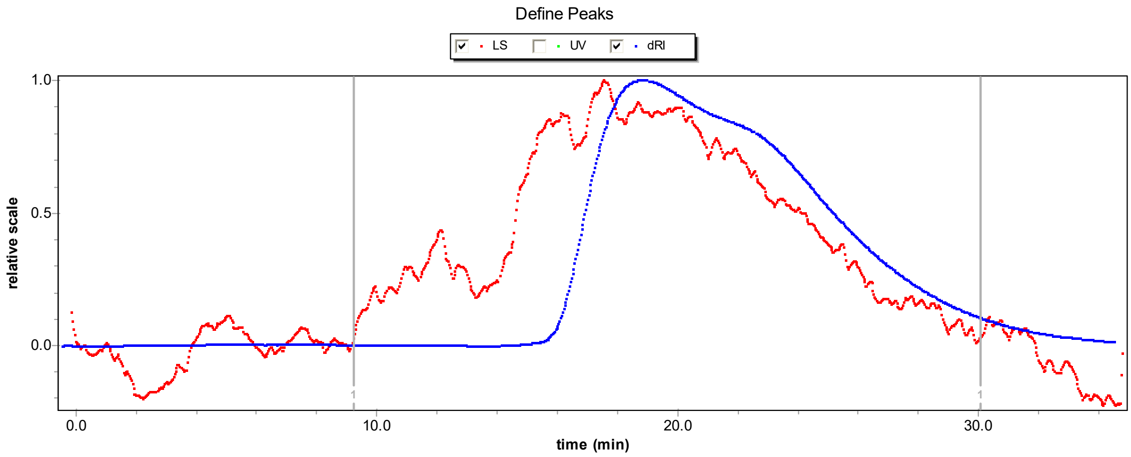

Figure S5. GPC chromatogram of carboxymethyl agar prepared using microwave-assisted method with DS of 0.48 (MAg2)

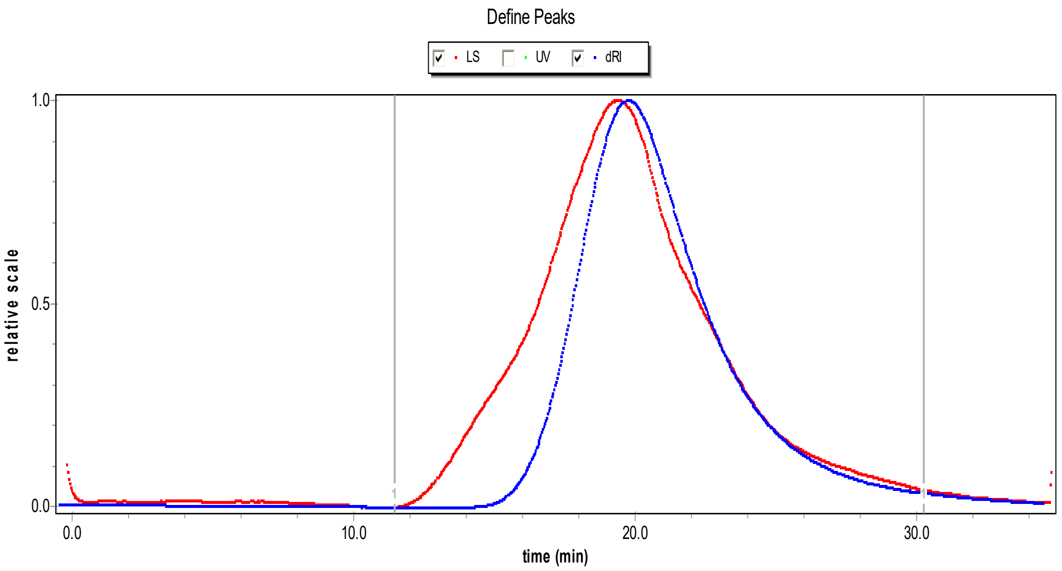

Figure S6. GPC chromatogram of dextran standards
